# Bleeding after percutaneous transhepatic biliary drainage due to arterial injury: A case study in patient with stable hemodynamic

**DOI:** 10.1016/j.radcr.2022.09.061

**Published:** 2022-10-12

**Authors:** Ira Widyaningtiyas, Hartono Yudi Sarastika, Harry Wahyudhy Utama

**Affiliations:** Department of Radiology, Faculty of Medicine Universitas Airlangga - Dr. Soetomo Academic General Hospital, Surabaya, Indonesia

**Keywords:** Percutaneous transhepatic biliary drainage, Cholangiography, Arteriography, Embolization

## Abstract

Percutaneous transhepatic biliary drainage (PTBD) is an effective procedure for correcting biliary obstructions. It can be performed under ultrasound and fluoroscopic equipment; however, it may entail serious complications, including bleeding, caused by arterial or venous injury. We present a 49-year-old man presented with a 1-month history of icterus, jaundice, dark urine, and right hypochondrial pain. MR imaging discovered a dilatation of the right intrahepatic bile duct due to obstruction by intrahepatic cholangiocarcinoma. PTBD procedure was performed in the right intrahepatic bile duct. After the pigtail drain device was inserted, the bile fluid color that came out from the pigtail turned sanguineous; nonetheless, the patient's hemodynamic was stable. Therefore, the second cholangiography was performed for evaluation. Some resistance was sensed during contrast injection into the bile duct, and the operator pushed the contrast media a little bit stronger and found a filling defect formed by a clot in the bile duct that suggested high suspicion of vessel injury. Although the patient's hemodynamics was still stable, the operator quickly decided to perform a hepatic arteriography procedure because bright red blood through the tube and a relatively rapid clot formed from the puncture point and distal drain, which were signs of hepatic artery injury. Hepatic arteriography confirmed the location of pseudoaneurysm caused by vessel trauma and arterio-intrahepatic bile duct fistulation. The embolization procedure was performed using PVA-300 into a ruptured hepatic artery branch through a microcatheter. Re-evaluation arteriography showed no pseudoaneurysm or arterio-intrahepatic bile duct fistulation after embolization.

## Introduction

Percutaneous transhepatic biliary drainage (PTBD) refers to a biliary obstruction correction procedure that is effective and readily accessible via ultrasound and fluoroscopic equipment [1]. It is a suggested treatment of choice for those who fail to undergo endoscopic retrograde [2], which can be caused by benign etiologies such as cholelithiasis, congenital stenosis, cystic dilations, and others, or malignancy such as cholangiocarcinoma, pancreatic cancer, and Ampulla of Vater cancer [1].

PTBD complications include bleeding, bile duct and hepatic artery or portal vein fistulae, pseudoaneurysms, bile leaks, risk of pneumothorax or hemothorax due to transpleural punctures occurring in 8.6%-22% of the procedures [2], and particularly, bleeding due to trauma on arterial or venous vessels [3]. The Society of Interventional Radiology has published a guideline indicating a major bleeding complications rate of 2.5% following PTBD, and a practice review is recommended should the cut-off rate of 5% is exceeded [4].

Hepatic artery bleeding following PTBD is marked by the presence of bright red and occasionally pulsatile blood in the tube, sometimes along with hemodynamic instability. Any evidence of abnormalities in the arteries nearby the percutaneous drain must be deemed the bleeding source [5], and such an abnormality should be meticulously catheterized and embellished. If feasible, the hepatic artery embolization should be done from the distal site to its proximal across the injury. Thereafter, the assessment of other potentially masked injuries entailed by prior procedures should be conducted using global arteriography [6,7].

## Case description

**Fig. 1:**
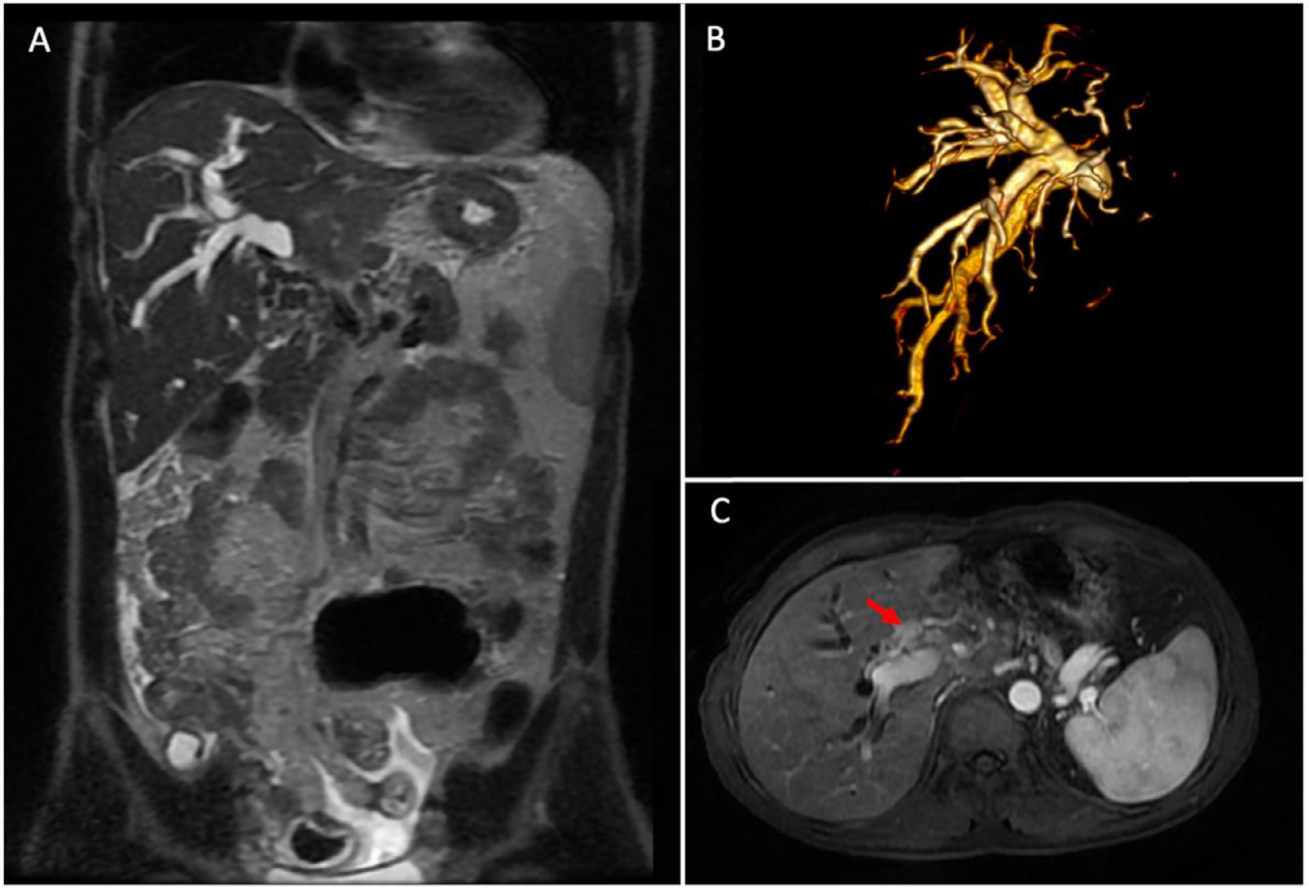
Abdominal MRI; (A) T2 FS sequence coronal view showed the dilatation followed by an abrupt termination of right intrahepatic bile duct; (B) 3D MRCP clearly showed the dilatation of right hepatic bile duct; (C) T1 FS contrast showed the contrast-enhanced mass (red arrow) that obstruct the right intrahepatic bile duct. © Department of Radiology, Dr. Soetomo General Academic Hospital, Medical Faculty of Universitas Airlangga, Surabaya, Indonesia (with permission).

A 49-year-old man presented with a 1-month history of yellowing of eye and skin, dark urine, and right hypochondrial pain. Examination using ultrasonography found the dilatation of right intrahepatic bile ducts. MR imaging found the dilatation of the right intrahepatic bile duct and no dilatation of the left intrahepatic bile duct. It also showed the contrast-enhanced mass that obstructs the bile duct (Fig. 1). The provisional diagnosis was cholangiocarcinoma. Blood test during admission showed an elevation in direct bilirubin level of 13.8 mg/dl, total bilirubin level of 18.6 mg/dl, platelet count of 216,000, and the prothrombin time and activated partial thromboplastin time value in the normal range.

**Fig. 2:**
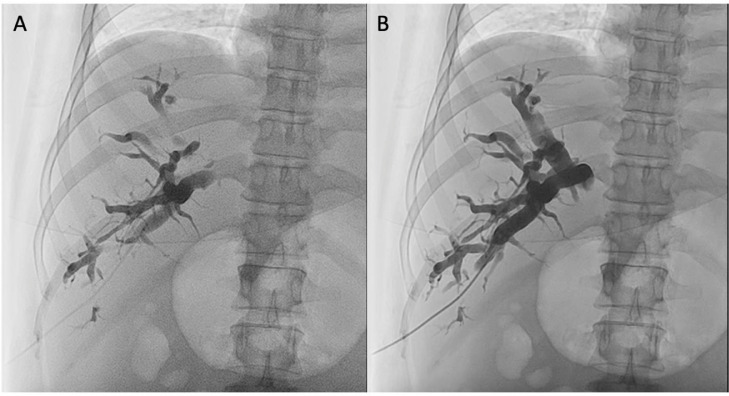
Percutaneous cholangiography; (A) introducer placed in large intrahepatic bile duct and contrast injected into the bile duct; (B) the contrast filled in the right intrahepatic bile duct and stopped. There was no contrast filling the left hepatic bile duct, common hepatic bile duct, or the common bile duct. No leaked contrast media was found in this image. © Department of Radiology, Dr. Soetomo General Academic Hospital, Medical Faculty of Universitas Airlangga, Surabaya, Indonesia (with permission).

Two days after admission, we performed a PTBD procedure in the right intrahepatic bile duct. In this procedure, we used *Boston* Introducer Kit, *Optitorque* pigtail size 6F, and Iopamiro as the contrast media. The choice of pigtail size 6F was adjusted to the most proximal and largest diameter of the bile duct that could be reached by the device. The procedure began with the puncture into the dilated bile duct. Using the ultrasound, we chose the nearest and largest bile duct dilatation. After the puncture and the bile came out from the puncture needle, we performed percutaneous cholangiography for further evaluation—the contrast filled in the dilated right intrahepatic bile duct and stopped. No contrast filled the left hepatic bile duct, common hepatic bile duct, or the common bile duct with no leakage of the contrast media (Fig. 2).

The next steps were inserting the pigtail drain device into the dilated intrahepatic bile duct. A pigtail was inserted and fixated inside the intrahepatic bile duct. However, the bile fluid color from the pigtail turns to sanguineous color soon after the drain installation. The patient's hemodynamics were stable, with a blood pressure of 120/70 mmHg and a heart rate of 80 beats per minute. We suspected a vessel injury and commenced the second cholangiography procedure with the pigtail still attached inside the bile duct.

**Fig. 3:**
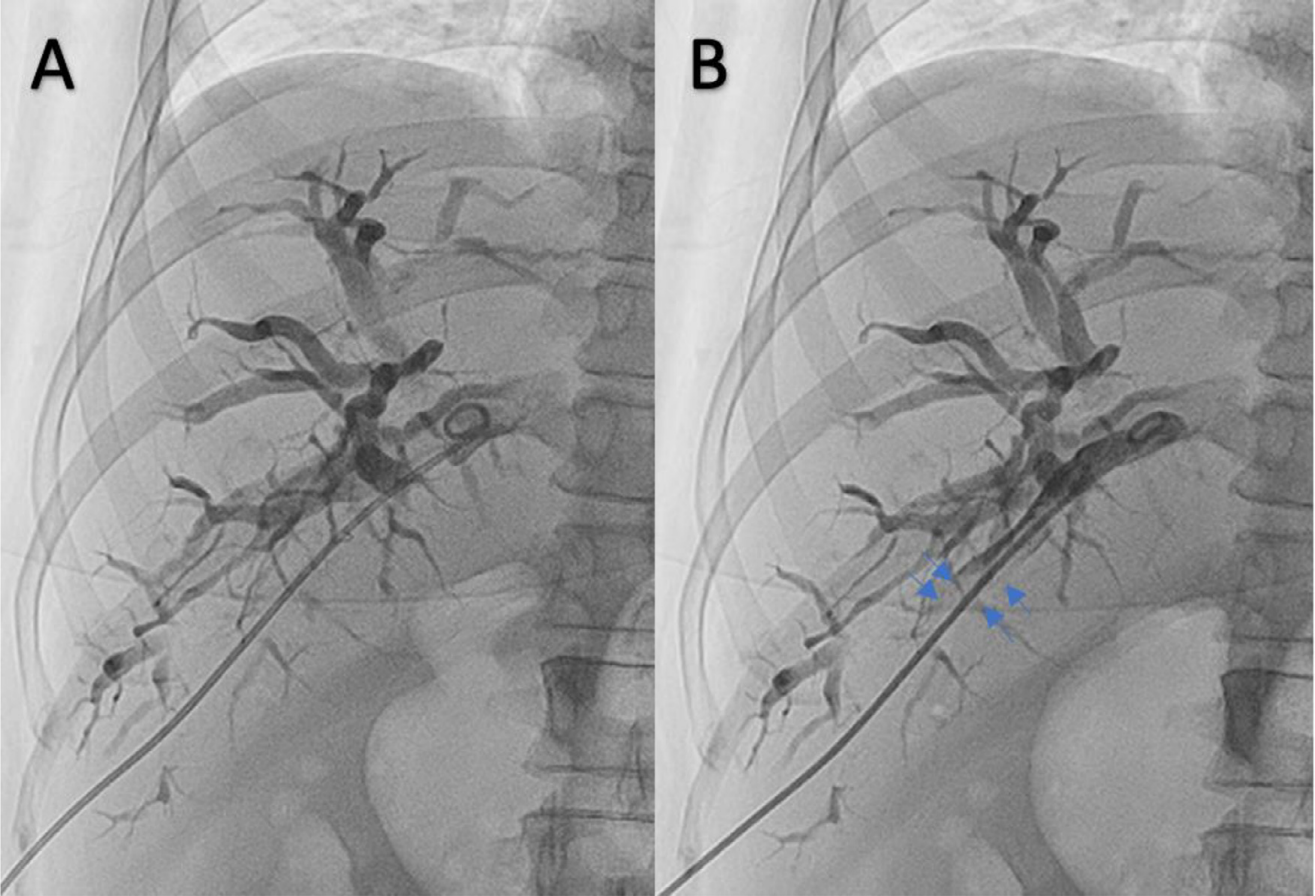
Pigtail drain device fixated inside the intrahepatic bile duct; (A) pigtail visualized inside intrahepatic bile duct; (B) contrast injected through the pigtail, there is filling defect in the intrahepatic bile duct (blue arrow). © Department of Radiology, Dr. Soetomo General Academic Hospital, Medical Faculty of Universitas Airlangga, Surabaya, Indonesia (with permission).

In this second cholangiography, the operator sensed resistance when pushing the contrast into the bile duct. We forced the contrast media slightly stronger and found a filling defect in the bile duct. The operator continued injecting the contrast media; however, the filling defect formed by a clot and injection resistance still existed (Fig. 3). A relatively rapid clot formation, which extended from the puncture point to the distal tube, showed arterial bleeding.

The interventionist decided to stop the contrast injection and observed the bile color that flowed out from the pigtail. During observation, the bile color from the pigtail drain appeared red and gradually changed to fresh red blood. This condition suggested a high suspicion of arterial injury. In this situation, we decided to perform hepatic arteriography to find the bleeding source or the vessel rupture location. Before starting this procedure, we re-evaluated the patient hemodynamic, revealing a blood pressure of 120/70 mmHg and heart rate of 89 beats per minute.

We started the arteriography procedure through the puncture at the common femoral artery (femoral approach) and guided the catheter until the right hepatic artery branch. Contrast media was then injected continuously through a catheter, and contrast filled the hepatic arteries, and some contrast pooled at the distal hepatic artery forming a pseudoaneurysm caused by vessel trauma.

**Fig. 4:**
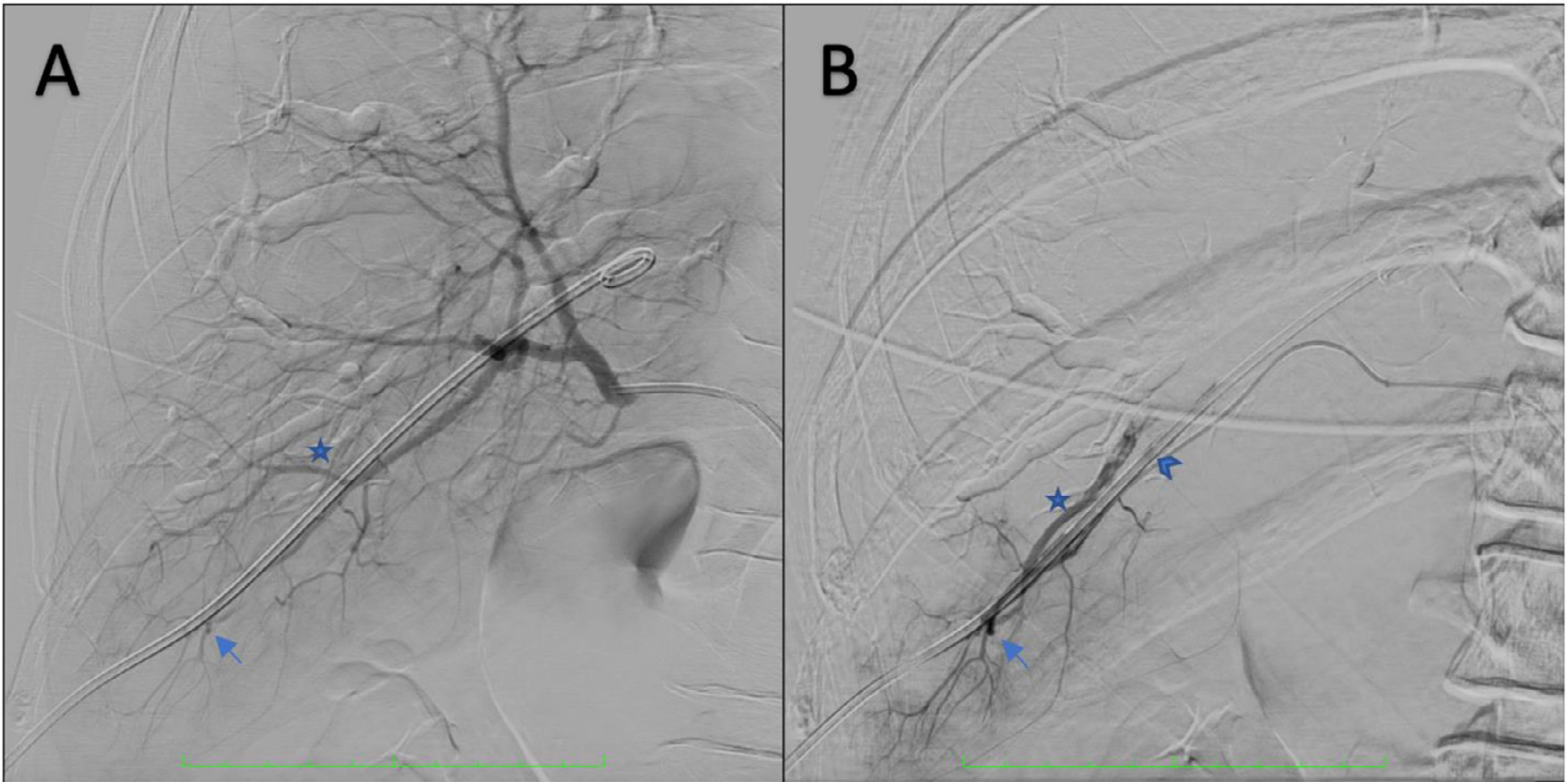
Hepatic arteriography; (A) contrast media was injected through a catheter and filled the hepatic arteries. A contrast pool was evident at the distal hepatic artery (blue arrow), a branch of the intrahepatic bile duct (star); (B) Selective arteriography using microcatheter. The contrast pool at the distal hepatic artery forming a pseudoaneurysm was strongly visible (arrow). There was also artery-intrahepatic bile duct fistulation as contrast fills around pigtail (arrowhead) drain and intrahepatic bile duct (star). © Department of Radiology, Dr. Soetomo General Academic Hospital, Medical Faculty of Universitas Airlangga, Surabaya, Indonesia (with permission).

Contrast also slowly filled the right intrahepatic bile duct branch due to artery-intrahepatic bile duct fistulation (Fig. 4).

**Fig. 5:**
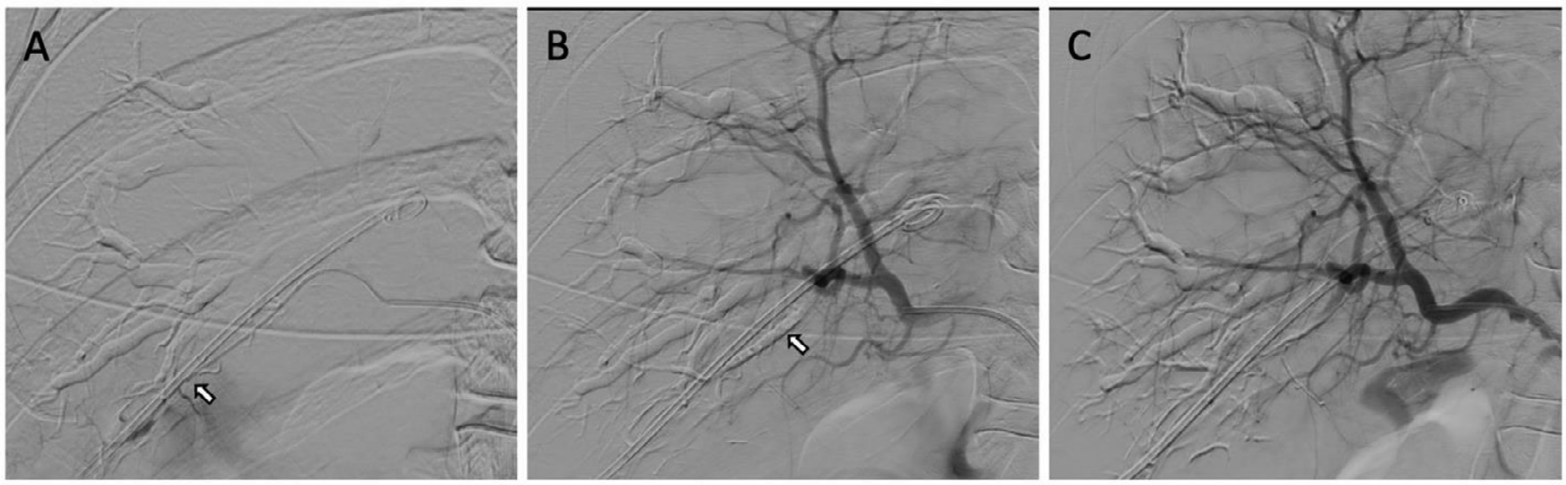
Embolization at hepatic artery branch; (A & B) PVA-300 was injected into the ruptured artery branch (white arrow). (C) Evaluation after embolization showed no pseudoaneurysm and artery-bile duct fistula. © Department of Radiology, Dr. Soetomo General Academic Hospital, Medical Faculty of Universitas Airlangga, Surabaya, Indonesia (with permission).

After the arterial rupture location was found, the embolization procedure was performed. PVA-300 was used to close the fistulation and stop the bleeding. PVA-300 was injected into a ruptured hepatic artery branch through a microcatheter. Then we evaluated the embolization area using contrast media, and no pseudoaneurysm, AV fistula, or artery-bile duct fistula was found (Fig. 5). Immediately after embolization, the bleeding came out from the drain suddenly stopped.

## Discussion

Bleeding complications succeeding the percutaneous transhepatic biliary drains may occur in the forms of hemothorax, hemoperitoneum, hemobilia, subcapsular hepatic hematoma, melena, and draining site bleeding [5], happening in roughly 2%-3% of patients undergoing transhepatic biliary drainage.

In this case study, the patient was admitted with cholangiocarcinoma. MR imaging found the dilatation followed by the abrupt termination of the right intrahepatic bile duct due to obstruction by enhancing mass, leading to a provisional diagnosis of cholangiocarcinoma. Our assessment was supported by a laboratory result indicating a direct bilirubin level of 13.8 mg/dl and a total bilirubin level of 18.6 mg/dl. Cholangiocarcinoma is one of the leading causes of obstructive jaundice and biliary strictures [8,9]. Hence, the PTBD procedure was selected to lower the blood bilirubin level.

The PTBD procedure was performed 2 days after the admission. We used ultrasound as guidance to identify the nearest and most significant bile duct dilatation and precise puncture location, passing the needle above the rib to avoid the intercostal vessel. Giurazza et al. [10] reported that using ultrasound for evaluating the biliary tree for PTBD was safe and effective with low severity and acceptable rate of complications. However, even though interventional radiologists widely adopt ultrasound imaging modality for multiple percutaneous approaches, it is still operator-dependent; therefore, the visualization accuracy and precise location rely on operator skill.

In order to avoid the intercostal vessels and nerves laying under each rib, the puncture site was placed above the rib. Also, the overlaying skin must be inspected for any dilated superficial veins [5]. After the needle puncture, we insert and set the introducer position. Bleeding was suspected if the bile fluid color from the introducer appeared sanguineous or frank blood. In our case, the bile color that comes out from the introducer was yellowish-green; therefore, we concluded that there was no vessel injury. Then, the cholangiography procedure was performed to assess the intrahepatic bile duct branch and the leak of the contrast media. In our case, no filling defect was discovered in the cholangiography procedure (Fig. 2).

During the process, the pigtail was inserted and fixated inside the intrahepatic bile duct. Shortly after, however, the bile fluid in the introducer turned into sanguineous color, suggesting a presence of vessel injury. Afterward, the operator performed the second cholangiography procedure with the pigtail still attached to the bile duct.

We found a constant filling defect in the intrahepatic bile duct, and some resistance was sensed when pushing the contrast into the bile duct. A filling defect inside the intrahepatic bile duct should be taken into consideration as there might be an arterial injury with a fistula formation toward the bile duct [5]. A filling defect indicated communication between the biliary system and a high-pressure vessel (an artery), provoked by a relatively rapid blood clot formation from the distal to the proximal tract. Gentle contrast injection was not powerful enough to reflux the contrast into the artery to visualize the bile duct [5] (Fig. 3).

Hepatic arterial bleeding presented with pulsatile, bright red blood in the drainage pouch, hemodynamic instability, and a notable decline in hematocrit levels [3]; therefore, many operators would directly conduct the arteriography to exclude a notable injury [6]. In our case, the patient's condition was relatively stable, and the operator decided to commence with hepatic arteriography using a femoral approach. Contrast media was injected through the catheter and filled the right hepatic artery and its branches, while some of the contrast pooled at the distal hepatic artery forming a pseudoaneurysm. Contrast also slowly filled the right intrahepatic bile duct branch due to artery-intrahepatic bile duct fistulation (Fig. 4). Some operators may perform arteriography first, especially if the patient is unstable [5]. Hepatic arteriography is performed most commonly from a femoral approach [5]. Arterial injuries include active extravasation, peripheral arterial truncation, arterial transection, pseudoaneurysms, arterioportal fistulae, and, rarely, arterio-hepatic vein fistula [5].

The embolization procedure was performed to forestall the bleeding. PVA-300 was used to occlude the fistulation and stop the bleeding, injected into a ruptured hepatic artery branch through a microcatheter (Fig. 5). Upon identifying an arterial injury, the involved vessel should be meticulously catheterized and embellished using coils. Hepatic artery embolization should be performed by inserting the coil into the artery from distal to proximal across the injury to prevent backward bleeding [7,11]. In this case, we used PVA due to coil unavailability in our center. Arteriography was also performed afterwards to exclude pseudoaneurysm, AV fistula, or artery-bile duct fistula. Global arteriography after embolization should be conducted to explore another injury that may have been masked after embolization of the main injury [7,11].

## Conclusions

PTBD is an effective procedure for correcting biliary obstructions; however, bleeding could occur as one of its complications in 2.5% of PTBD patients. Hence, it is essential to recognize the early sign and symptoms of the bleeding from physical status and cholangiography. Identifying the signposts of bleeding due to arterial injury with stable hemodynamics leads to faster embolization treatment in a more comfortable environment without worrying about the patient's hemodynamic condition. Hepatic arteriography should be performed once vessel injury is suspected from the transhepatic cholangiography. An embolization procedure was performed to close the fistulation and stop the bleeding, with arteriography performed following the procedure to explore another injury that may have been unmasked after the embolization of the main injury.

## Ethic committee approval

This study has met the ethical principle and has already obtained approval from Research Ethics Committee from Dr. Soetomo General Hospital, Surabaya.

## Patient consent

Written informed consent was obtained from the patient for the publication of this case report.
